# Using Satellite Tracking and Isotopic Information to Characterize the Impact of South American Sea Lions on Salmonid Aquaculture in Southern Chile

**DOI:** 10.1371/journal.pone.0134926

**Published:** 2015-08-26

**Authors:** Maritza Sepúlveda, Seth D. Newsome, Guido Pavez, Doris Oliva, Daniel P. Costa, Luis A. Hückstädt

**Affiliations:** 1 Centro de Investigación y Gestión de Recursos Naturales (CIGREN), Instituto de Biología, Facultad de Ciencias, Universidad de Valparaíso, Valparaíso, Chile; 2 Biology Department, University of New Mexico, Albuquerque, United States of America; 3 University of California Santa Cruz, Ecology and Evolutionary Biology, Santa Cruz, California, United States of America; Institut Pluridisciplinaire Hubert Curien, FRANCE

## Abstract

Apex marine predators alter their foraging behavior in response to spatial and/or seasonal changes in natural prey distribution and abundance. However, few studies have identified the impacts of aquaculture that represents a spatially and temporally predictable and abundant resource on their foraging behavior. Using satellite telemetry and stable isotope analysis we examined the degree of spatial overlap between the South American sea lion (SASL) and salmon farms, and quantify the amount of native prey versus farmed salmonids in SASL diets. We instrumented eight SASL individuals with SRDL-GPS tags. Vibrissae, hair and skin samples were collected for δ^13^C and δ^15^N analyses from five of the tagged individuals and from four males captured in a haul-out located adjacent to salmon farms. Tracking results showed that almost all the foraging areas of SASL are within close proximity to salmon farms. The most important prey for the individuals analyzed was farmed salmonids, with an estimated median (±SD) contribution of 19.7 ± 13.5‰ and 15.3 ± 9.6‰ for hair and skin, respectively. Using vibrissae as a temporal record of diet for each individual, we observed a remarkable switch in diet composition in two SASL, from farmed salmonids to pelagic fishes, which coincided with the decrease of salmon production due to the infectious salmon anemia virus that affected salmon farms in Chile at the end of 2008. Our study demonstrates the usefulness of integrating stable isotope derived dietary data with movement patterns to characterize the impacts of a non-native prey on the foraging ecology of an apex marine predator, providing important applied implications in situations where interactions between aquaculture and wildlife are common.

## Introduction

The diet composition of generalist and opportunistic predators is expected to shift temporally and/or geographically in response to changes in prey distribution and availability [1], [2], allowing predators to exploit prey aggregations that are presumably easier to capture when they occur at high density [3], or switching among prey in accordance with its abundance [4–6]. However, if prey can predictably be found and captured in a particular location, it is anticipated that predators will show corresponding changes in foraging behavior and spatial distribution in response to that predictability [1], [7–9]. This is particularly evident in the interaction between domestic animals and terrestrial predators, where the high density of livestock, whose predictability, vulnerability and containment in enclosures stimulate changes in foraging behavior and spatial distribution of predators [10–12].

For pinnipeds (seals, sea lions, and walrus), the acquisition of food is a major challenge because many species move between breeding or hauling-out sites on land that can be 100s or 1000s of kilometers from their aquatic foraging grounds. In dynamic, heterogeneous environments such as ocean ecosystems, several studies have shown how individuals change their foraging behavior in response to spatial and/or seasonal changes in natural prey distribution and abundance [13–15], although for Australian sea lions (*Neophoca cinerea*) a similar foraging pattern was previously observed despite spatial and temporal variability in oceanographic conditions [16]. There are only a few studies of pinniped foraging ecology, however, that identify the impacts of prey species that are confined to small enclosures in the ocean, such as the conditions associated with salmonid aquaculture [17–18].

The presence of cultivated salmon in small pens at high density inevitably constitutes a powerful food attractant to opportunistic coastal marine mammals, seabirds, and fish that normally feed on native fish stocks [19]. Among these predators, pinnipeds are among the most troublesome because they have plastic feeding strategies and individuals can learn to exploit situations where salmon are concentrated and vulnerable, which may result in significant economic losses to fish farm operators [17–20]. In Chile, a strong operational interaction between the South American sea lion (SASL, *Otaria byronia*) and the salmon farming industry has been previously described [18], [20]. This high interaction could be explained by a combination of high abundance of SASL in southern Chile (~44,000 individuals) [21], its generalist and opportunistic diet primarily based on fish [22], and the presence of >300 salmon farming installations in a relatively small region. However, little is known about whether, and how, SASL modify their foraging behavior in response to the high predictability and availability of this non-native prey source.

The recent development and adoption of two complimentary technologies–GPS telemetry and stable isotope analysis–has enabled ecologists to link high-resolution movement data with dietary information. With the development of Fastloc-GPS telemetry tags, the location and movement patterns of marine mammals can be tracked at sea with high accuracy (<40 m) and for extended periods of time [23], thereby allowing for an assessment of spatial overlap between prey and predator [24]. The use of cost-effective stable isotope analysis (SIA) has provided greater insights into foraging ecology across a range of elusive marine mammals [16], [25–26] for which dietary information gleaned from traditional techniques (stomach or scat contents) is limited. Thus, the study of individual foraging strategies in pinnipeds may greatly benefit from combining telemetry data with dietary information derived from stable isotope analysis [27, 28]. In contrast to traditional proxies used to study marine mammal diet, SIA can quantify variation in diet composition and habitat use at the individual and population level [26], [29–31]. Carbon (δ^13^C) and nitrogen (δ^15^N) isotope values of a consumer are related to that of its diet, but are offset by a predictable amount due to isotopic discrimination that occurs during resource assimilation and metabolism [32–33]. δ^15^N values increase by ~3–5‰ per trophic step and are typically used to quantify trophic level, while δ^13^C values are generally used to assess habitat use in marine contexts [26]. Since isotopic incorporation rates vary among metabolically active tissues (*e*.*g*., muscle or blood), this method is ideal for identifying dietary variation over a range of time scales [34–36]. Isotopic analysis of metabolically inert but continuously growing tissues (*e*.*g*., vibrissae or nails) can be serially sub-sampled to provide a longitudinal record of foraging information at the individual level [28–30], [37]. Specifically, SIA of vibrissae has been used to identify broad temporal and ontogenetic variation in foraging location and trophic level in several species of pinnipeds [15], [28–29], [37–42].

The objective of this study is to analyze how the foraging behavior of a generalist and opportunistic marine predator is influenced by a novel and abundant prey source in the form of farmed salmonids. To do this we combined satellite telemetry data with δ^13^C and δ^15^N stable isotope analysis of hair, skin, and vibrissae to examine the degree of spatial overlap between SASL and salmon farms, and quantify the amount of native prey versus farmed salmonids in SASL diets. If salmonids are an important prey item for SASL we predicted that: (1) SASL feeding areas would frequently overlap with the locations of salmon farms, and (2) that farmed salmonids would be well represented in the diet of SASL. Because salmonid escapes have detrimental impacts on native fishes due to predation and competition [43], our results represent an important step in understanding the potential role of SASL as a natural regulator of this non-native fish in Chile.

## Materials and Methods

### Ethics statement

All SASL handling and tagging procedures were authorized by Subsecretaría de Pesca Permits N° 2799/2008 and 1737/2010, and approved by the Bioethical Committee at the Universidad de Valparaíso and by the Institutional Animal Care and Use Committee at University of California Santa Cruz.

### Field site and general procedure

**Fig 1:**
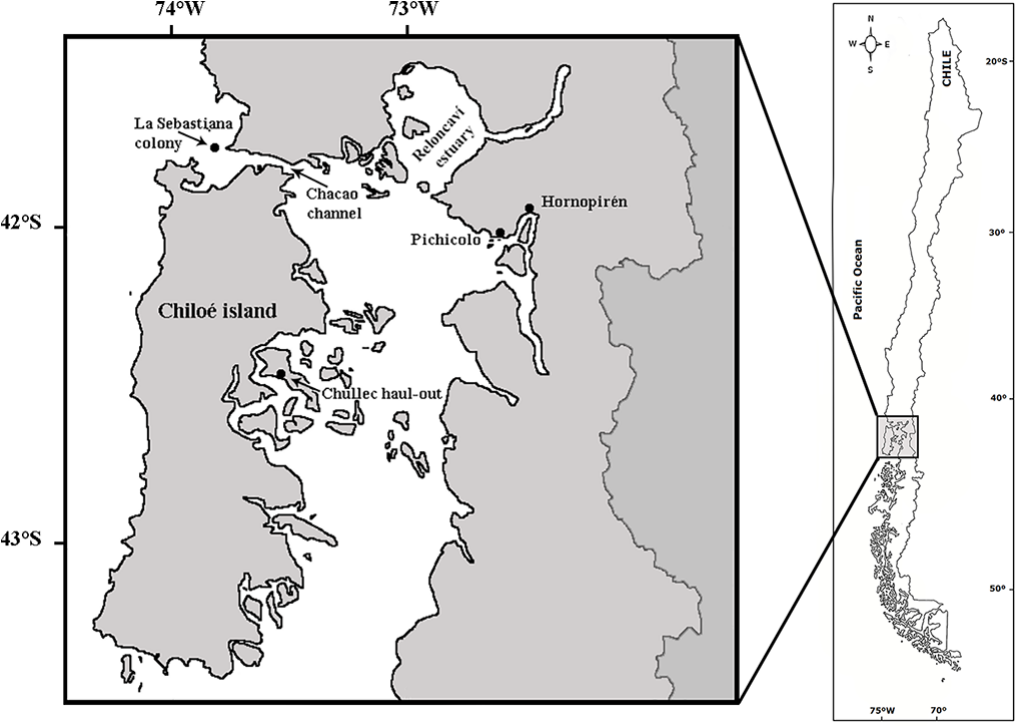
Sites of captures and locations used by eight South American sea lions (*Otaria byronia*) instrumented in southern Chile during their foraging trips.

We captured eight SASL (2 males and 6 females) in July 2009 (n = 4) and June 2010 (n = 4) on the coast in front of Calbuco (41°48’S; 73°08’W) (n = 7, CA-01 to CA-07) and Pichicolo (42°01’S; 72°35’W) (n = 1, PI-01), southern Chile (Fig 1). SASL were captured as they approached commercial purse-seine vessels to forage during sardine fishing using a hoop net in the water, brought on board the vessel and then transported to shore as described in [44]. During capture and transit to the handling site animals were constantly monitored by a veterinarian. Once on shore, sea lions were anaesthetized with isofluorane gas (0.5 to 2.5%) and oxygen via a portable field gas anesthesia machine. The gas was administered through a cone shaped mask and then with an endotracheal tube. Breathing rate and tactile response, as well as blood O_2_ concentration and CO_2_ saturation were monitored continually and used to assess animal condition during anesthesia. Individuals were instrumented with Sea Mammal Research Unit-Satellite Relay Data Logger (SRDL) GPS tags (University of St. Andrews, Scotland), glued to the dorsal pelage between the shoulder blades using 5-min marine epoxy. Standard length and axillary girth measurements were recorded, and animals were weighed before release using a 500 kg (±0.1 kg) digital scale.

For stable isotope analysis, we collected vibrissa samples from five of the eight individuals (CA-01 to CA-04, and PI-01) that were instrumented with satellite tags. We complemented this dataset with vibrissae from other four males (three subadults and one juvenile; CH-01 to CH-04) captured in the Chullec haul-out (42°28’S; 73°33’W) on the inner (eastern) coast of Chiloe Island (Fig 1). During capture, the longest vibrissa of each animal was selected and collected for analysis by plucking it from the root with a pair of pliers or forceps. Samples were washed with distilled water and detergent and allowed to air dry, and then rinsed in an ultrasonic bath with petroleum ether for 15 min to remove surface contaminants.

Vibrissae were then measured to the nearest cm, and sub-sampled using a length-based strategy in which a ~0.5 mg segment was collected every ~0.33 cm along the vibrissa from the base to the tip; this approach produced a mean (±SD) of 37 ± 8 segments from each vibrissa. To estimate the time period represented in each segment, and in the absence of data on SASL vibrissae growth rates, we used a rate of 0.16 mm d^-1^, based on published data for other otariids, including California sea lion *Zalophus californianus* (0.16 mm d^-1^) [45], Steller sea lions *Eumetopias jubatus* (0.10–0.14 mm d^-1^) [38], and Antarctic fur seals *Arctocephalus gazella* (0.11–0.16 mm d^-1^) [37].

Hair and skin samples were also collected from the captured animals from both Calbuco and Chullec while they were under anesthesia. Skin was obtained by cutting a small sample of pectoral flipper (3 x 3 mm^2^). Samples were then stored in sterile Eppendorf tubes and maintained on ice in the field. Samples were put in heat-sealed Ankom filter bags and placed in a universal oven and dried at 60°C for 72 h, and minced with a scalpel. Finally, lipids were extracted from skin samples with petroleum ether in a Soxhlet extractor for 2 h, and then stored in 2.0 mL screw cap microtubes (Biologix Research Co., USA).

Samples from 16 potential native prey species and three farmed salmonid species were also collected. Samples were collected from August 2009 to January 2010, thus representing a temporal window of ~6 months. Native prey samples were collected directly from minor-scale fisheries landings, whereas salmonids were obtained directly from salmon farms. Native prey species were defined as potential prey items based on: (1) a previous study of diet composition of SASL in the study area [46], and (2) information of species consumed by sea lions in interactions with artisanal fisheries in the study area [47], [48]. The 16 native prey species collected for stable isotope analysis represented about two-thirds (66.7%) of the potential prey species available to sea lions [49]. The total length range of analyzed prey was within the ranges reported in other studies that examined the diet of SASL in other regions of the country [50–51]. Only mature individuals from each prey species were selected. A section of ~1 cm^3^ of muscle was taken from each prey using a scalpel, stored in Eppendorf tubes and frozen. In the laboratory, samples were dried at 60°C for 72 h, homogenized with mortar and pestle, and lipid extracted using the same technique described above.

### Tracking analysis and spatial interaction with salmon farms

SRDL-GPS tags collected and stored data on location and diving behavior of each animal. Each tag was programmed to transmit data via the ARGOS satellite system using a repetition rate of 30–60 s [52]. Location data were obtained using on board Fastloc-GPS technology, which is dependent on the number of satellites used to calculate the location [53], and as a result some erroneous locations are possible. In order to eliminate such data, we ran the sea lion tracking data through a simple speed filter using a conservative threshold of 5.5 m s^-1^ [44]. Finally, tracking data were linearly interpolated at a regular time interval (4 h). Due to the intricate coastline of our study site (Fig 1), we implemented a land mask for all spatial calculations, so that our results were restricted to at sea locations only. All spatial analyses were conducted in ArcGIS 10 (ESRI, Redlands, CA, USA).

To address the question of space use by SASL, three complementary approaches were applied: (1) a home range kernel (Gaussian kernel, bandwidth determined using the plugin method) in order to identify animal high usage areas [54], (2) individual-based home range kernel analysis, to evaluate among individual habitat usage [55], and (3) the proportion of time-at-sea that individual SASL spent within a radius of 1, 5, and 10 km from salmon farms. These distances were included in our analysis considering the accuracy of the GPS, the average distance between salmon farms (3 to 6 km), and the distance that a sea lion could cover in the interval of time between successive real locations successfully obtained by the SRDL-GPS tag and transmitted over the Argos system. Salmon farm locations were obtained from the Instituto Tecnológico del Salmón (INTESAL) database, which is part of the Asociación de la Industria del Salmón, A.G. SalmonChile (http://www.salmonchile.cl/). Kernel home range analyses were performed in Geospatial Modelling Environment [55].

### Stable isotopes analysis and Bayesian mixing model

Approximately 0.5 ± 0.05 mg of vibrissae, hair and skin samples of sea lions and muscle samples from potential prey were sealed into tin capsules for isotopic analysis. δ^13^C and δ^15^N values were measured using a Carlo-Erba NC2500 (Milan, Italy) or Costech 4010 (Valencia, California, USA) Elemental Analyzer interfaced with a Finnigan Delta Plus XL mass spectrometer (Waltham, Massachusetts, USA) at the University of Wyoming Stable Isotope Facility (Laramie, WY, USA). Isotopic results are expressed as δ values, δ^13^C or δ^15^N = 1000 x [(*R*_smpl_—*R*_stnd_)/*R*_stnd_], where *R*_smpl_ and *R*_stnd_ are the ^13^C/^12^C or ^15^N/^14^N ratios of the sample and standard, respectively. The units for δ-values are expressed as parts per mil (‰). The internationally accepted standards are Vienna-Pee Dee Belemnite limestone (VPDB) for carbon and atmospheric N_2_ for nitrogen. Repeated analysis of internal proteinaceous reference materials resulted in analytical precision of < 0.2‰ for both δ^13^C and δ^15^N values.

We used a Bayesian mixing model MixSIR v1.0 to determine the relative contributions of different prey groups in each individual sea lion’s diet [56]. Inputs into the model include the mean isotope values of potential prey and trophic discrimination factors (TDF) of tissues analyzed to estimate the probability distributions of the proportional contribution of each prey item (source) in the predator diet (mixture). Additionally, this model incorporates sources of uncertainty in both prey values and TDFs as standard deviation. Because isotopic mixing models cannot differentiate between the contributions of different prey unless they have significantly different isotope values [56–58], we pooled the 19 potential prey species into seven functional groups (Table 1). These groups were defined according to both similarity in the isotopic values and ecological function of prey items. Isotopic values of SASL were entered individually (for hair and skin), while isotope values of the prey groups were entered as means (±SD). For vibrissae, each individual segment was analyzed separately to provide an estimate of temporal variation in diet composition within each individual.

**Table 1:** Prey composition and δ^13^C and δ^15^N isotope values for each of the prey species and groups considered in MixSIR analysis. Values are shown as mean ± SD.

Group name	Species	*n*	δ^13^C	δ^15^N
Farmed salmonids	*Oncorhynchus mykiss*	19	-17.4 ± 0.8	12.9 ± 1.1
	*Oncorhynchus kisutch*	3	-16.5 ± 0.1	13.0 ± 0.6
	*Salmo salar*	1	-17.3 ± 0.0	13.9 ± 0.0
	Total	23	-17.3 ± 0.8	13.0 ± 1.1
Pelagic cephalopods	*Dosidicus gigas*	2	-16.2 ± 0.1	16.3 ± 0.2
Small pelagic fish	*Sprattus fuegensis*	12	-15.6 ± 0.4	15.3 ± 0.3
Demersal fish	*Seriolella caerulea*	2	-14.7 ± 0.4	19.2 ± 0.3
	*Merluccius australis*	14	-14.7 ± 0.5	18.0 ± 1.1
	*Helicolenus lengerichi*	5	-14.5 ± 0.3	17.3 ± 1.0
	*Genypterus* sp.	11	-14.2 ± 0.3	17.7 ± 0.9
	*Salilota australis*	6	-14.2 ± 0.2	17.8 ± 0.5
	*Mustelus mento*	4	-14.0 ± 0.7	18.6 ± 2.0
	*Callorhynchus callorhynchus*	6	-14.1 ± 0.1	19.0 ± 0.8
	Total	48	-14.4 ± 0.4	18.1 ± 1.1
Pelagic fish	*Thyrsites atun*	7	-15.1 ± 0.3	18.1 ± 1.0
	*Trachurus murphyi*	3	-15.1 ± 0.4	17.3 ± 0.4
	Total	10	-15.1 ± 0.3	17.8 ± 0.9
Benthic fish	Paralichthyidae	7	-14.7 ± 0.6	16.3 ± 1.4
	*Paralabrax humeralis*	9	-14.2 ± 0.9	16.2 ± 0.5
	*Eleginops maclovinus*	12	-13.7 ± 1.2	15.6 ± 1.0
	Total	28	-14.1 ± 1.0	15.9 ± 1.0
Benthic crustaceans	*Homalaspis plana*	2	-13.6 ± 0.4	14.3 ± 0.1
	*Cancer setosus*	1	-12.6 ± 0.0	15.5 ± 0.0
	Total	3	-13.3 ± 0.6	14.7 ± 0.7

At present, there are no published estimates of δ^13^C or δ^15^N TDFs for SASL. We used tissue-diet TDF values of 2.5 ± 0.5‰ for δ^13^C and 3.5 ± 0.5‰ for δ^15^N in the mixing model for vibrissae and hair, because these tissues are similar in biochemical composition [26], [34], [59]. For skin we used TDFs values of 2.5 ± 0.5‰ and 3.0 ± 0.5‰ for δ^13^C and δ^15^N, respectively [9]. These values are similar to the range reported in vibrissae of the sea otter *Enhydra lutris nereis* [59], as well as within the range of mean enrichment factors estimated for pinniped tissues [34], [60]. Because TDF can vary depending on prey type, prey quality, and the general metabolic pathway(s) of consumers [30], [59], we used a standard deviation of 0.5‰ in all the TDFs values to incorporate uncertainty in the estimation of prey proportions [56]. We used non-informative source contribution priors and a minimum of 10^6^ iterations when running the isotope mixing model. Results for the posterior contributions of prey groups to diet are expressed as a median value with standard deviation (SD).

The isotope values between tissues and between locations were compared using one-way analyses of variance (ANOVA). For statistical comparisons that did not fulfill the requirement of normality (Shapiro-Wilks test) and homogeneity of variances (Levene test), we used non-parametric Kruskal-Wallis and Mann-Whitney tests. All statistical analyses were performed using the software Statistica 8.0. Significance level was set at 95% for all statistical tests. Results are reported as mean ± SD, unless otherwise stated.

## Results

### SASL morphometrics and tag performance

Mean body length and body mass of the eight instrumented individuals were 156.1 ± 15.9 cm and 91.3 ± 25.3 kg, respectively (Table 2). Tracking records varied in duration from 7.8 to 137 d (mean = 60.9 ± 43.3 d), rendering a total of 4323 ± 237 GPS filtered locations for each individual.

**Table 2:** Morphometric and trip metric of the South American sea lion (*Otaria byronia*) (values expressed as mean ± SD). ID: identification number.

ID	Sex	Body length (cm)	Mass (Kg)	# foraging trips	Mean trip duration (days)	Total # days recorded
CA-01	Female	165	110.2	16	5.5 ± 3.1	103.7
CA-02	Female	146	68.4	8	1.7 ± 1.3	24.7
CA-03	Female	172	110.4	35	3.0 ± 1.9	137.0
CA-04	Male	129	52.8	11	1.9 ± 1.1	30.1
CA-05	Female	169	101.8	18	2.0 ± 1.8	45.8
CA-06	Female	147	85.1	3	1.7 ± 1.1	7.8
CA-07	Female	173	127.6	21	3.1 ± 1.6	74.9
PI-01	Male	148	73.9	48	0.9 ± 0.9	63.3

### Habitat utilization and foraging trips

**Fig 2:**
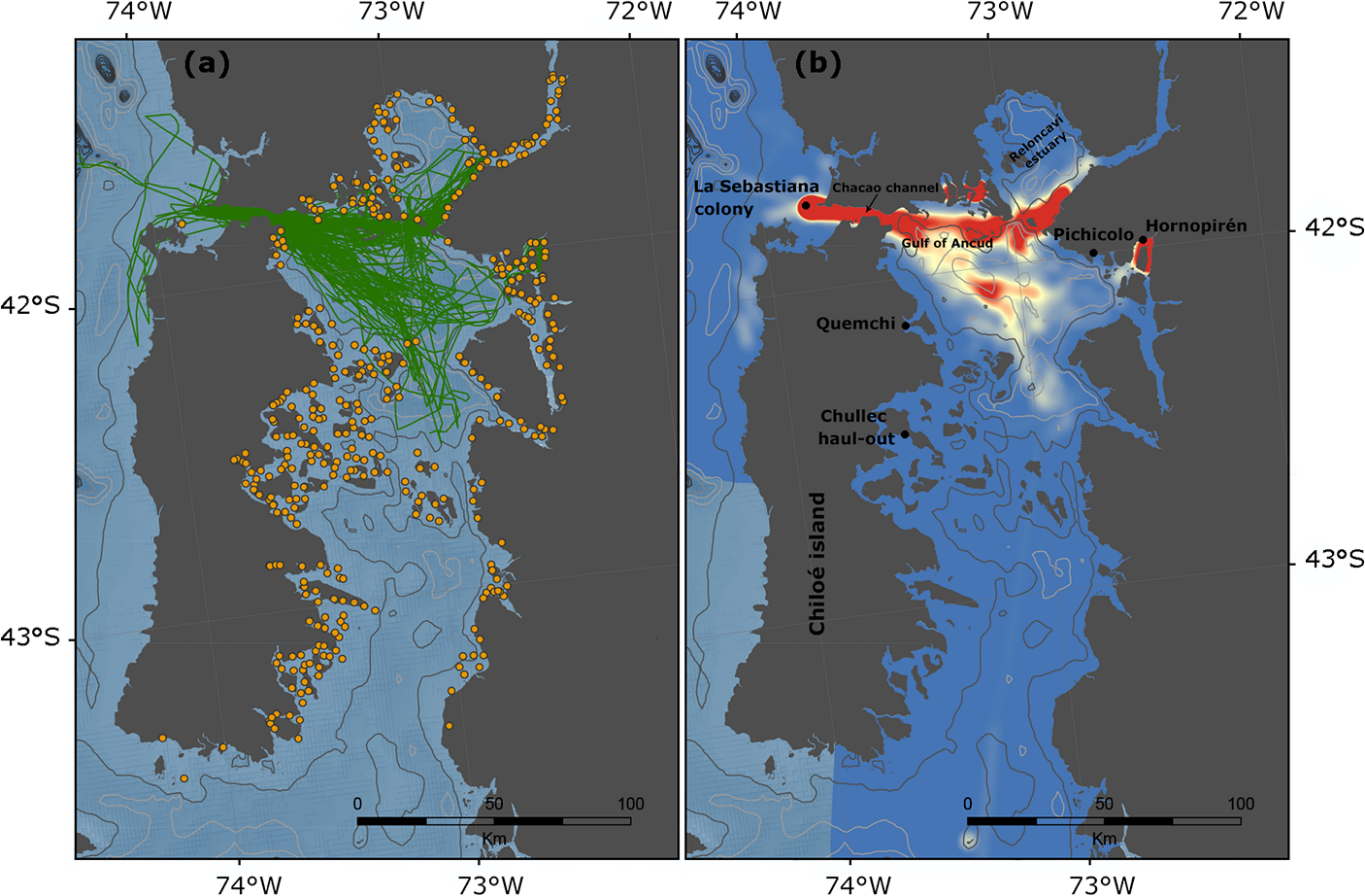
(a) GPS locations (green lines) from eight South American sea lions (*Otaria byronia*) instrumented in southern Chile during the austral winter of 2009 and 2010. Yellow dots represent the spatial distribution of salmon farms in the study area. (b) Kernel density estimates of sea lion location data. Red color represents higher use by sea lions

A total of 160 foraging trips were recorded, with a mean duration of 2.5 ± 1.4 d, whereas haul-outs ranged between 0.48 and 1.67 d (N = 142 haul-out periods) (Table 2). Most foraging trips were concentrated between La Sebastiana breeding colony (41°45’S; 73°48’W) and the inner waters of Chiloe Island (Fig 2A). The maximum distance travelled from La Sebastiana ranged between 74.6 and 127.1 km, whereas the total distance travelled (sum of the entire trips for each sea lion) ranged from 752 to 3974 km. The only exception was a subadult male (PI-01), captured in the inner waters of Chiloe Island (Pichicolo). This individual remained very close to the capture site colony through the duration of its tracking records. The kernel home range analysis allowed the identification of at least four hot spots of foraging behavior (defined as the 50% Utilization Distribution contour): (1) one in the Gulf of Ancud, located north of Chiloé Island; (2) a second located at the mouth of Reloncaví estuary; (3) a third located in the inner coast of Chiloe adjacent to Quemchi, and (4) a fourth in the waters around Pichicolo and Hornopirén. All of these hot spots were located in the inner coast (Fig 2B).

### Spatial interaction with salmon farms

We calculated the proportion of time that SASL spent within a radius of 1, 5, and 10 km from salmon farms (Table 3). On average, sea lions spent 3.5% of their time-at-sea within 1 km or less from the fish farms. However, this percentage increased to 41.7% and 69.1% when we considered the time spent within 5 and 10 km of the salmon farms, respectively. The exception was the individual PI-01who spent 98% of its time-at-sea within a 5 km radius of salmon farms, and over 22% of its time within 1 km.

**Table 3:** Proportion of time-at-sea (%) that SASL are in a radius of 1, 5 and 10km from a salmon farm in southern Chile, and median contribution (± SD) of farmed salmon to the diet of SASLs by hair and skin data.

ID	Proportion of time at-sea close to salmon farms (%)			Median contribution of farmed salmon	
	1 km	5 km	10 km	Hair	Skin
CA-01	0.9	12.0	43.7	20.0	6.2
CA-02	0.5	14.5	67.0	20.1	-
CA-03	4.9	51.0	82.1	-	7.7
CA-04	8.5	39.0	74.4	47.6	10.6
CA-05	1.3	14.7	39.7	18.7	17.4
CA-06	2.1	15.2	65.2	34.9	26.5
CA-07	0.1	50.3	66.8	27.6	8.5
PI-01	22.8	97.9	99.4	8.1	18.6
All	3.5	41.7	69.1	25.3	13.6

### Stable isotopes analyses

**Fig 3:**
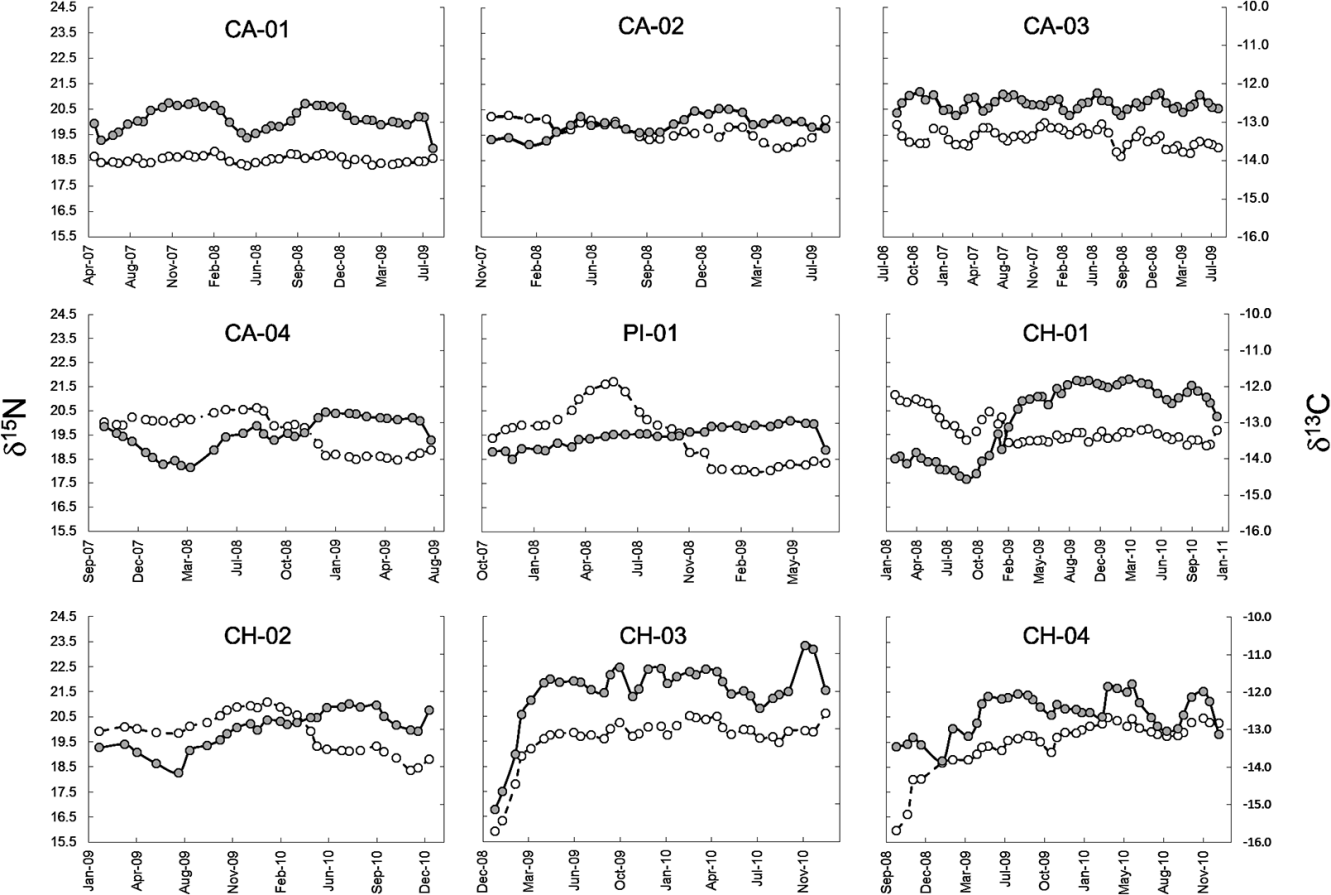
Variation of δ^13^C (gray dots) and δ^15^N (white dots) values along vibrissae of South American sea lions. Individual IDs are provided in the top of each panel.

The collected samples of vibrissae (N = 9) ranged from a length of 10.5 to 17.8 cm (mean: 13.2 ± 2.7 cm). From these, a total of 330 vibrissae segments were analyzed for δ^13^C and δ^15^N. Isotopic values of vibrissae showed relatively low variability (range <2‰) in mean δ^13^C and δ^15^N values among the nine individuals analyzed. No clear pattern was observed in δ^13^C or δ^15^N along vibrissae among individuals (Fig 3). SASL from Chullec showed higher within-vibrissae variability in δ^13^C values varying from 1.9‰ in CH-02 to 4.3‰ in CH-03. In contrast, individuals from Calbuco showed a low range of δ^13^C values, from 0.9‰ in CA-02 to 1.5‰ in CA-04 (Fig 3). A similar pattern was found in δ^15^N values, with a within-vibrissae range of 2.1‰ (CH-01) to 4.5‰ (CH-04) in sea lions from Chullec, whereas sea lions from Calbuco had within-vibrissae ranges of 0.5‰ (CA-01) to 1.3‰ (CA-03) (Fig 3). The only exception to this pattern was PI-01, which had a within-vibrissae range of 6.5‰ in δ^15^N.

δ^13^C and δ^15^N values of each individual for hair and skin tissues are shown in S1 Table. No significant differences were found in isotope values between hair and skin, neither for δ^13^C (*U* = 49.0, *P* > 0.05) nor for δ^15^N (*F*_1,20_ = 0.13, *P* > 0.05). There were no differences in δ^13^C values between Calbuco and Chullec in mean hair (*F*_1,8_ = 2.63, *P* = 0.143) or skin (*F*_1,7_ = 1.57, *P* = 0.250) values. Similarly, no difference was found in δ^15^N values for skin (*F*_1,7_ = 3.207, *P* = 0.116). The only exception was δ^15^N in hair, in which sea lions from Chullec showed higher values than sea lions from Calbuco (*F*_1,8_ = 8.90, *P* = 0.018).

### Diet composition

**Fig 4:**
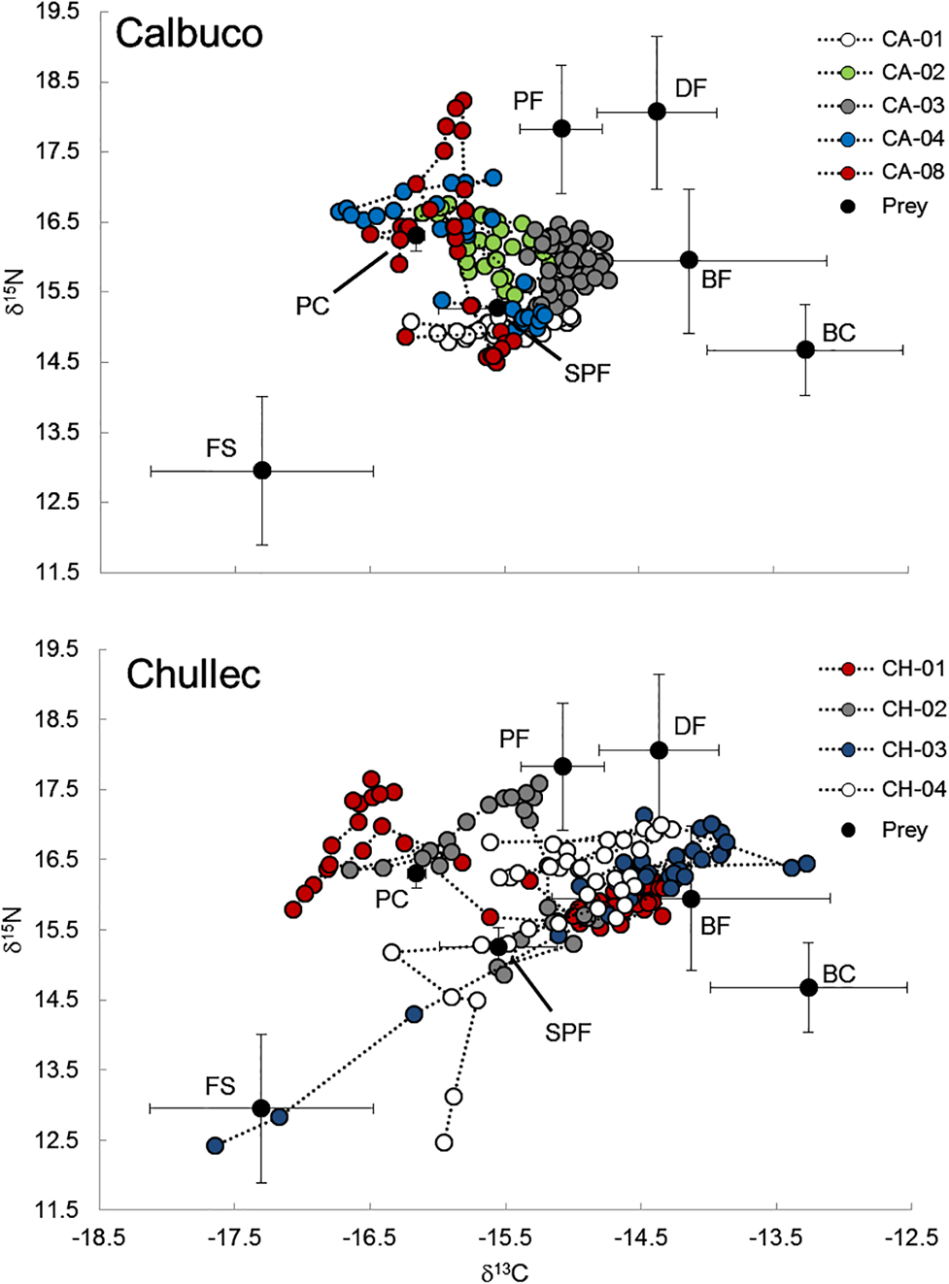
δ^13^C plotted against δ^15^N values for each segment along the length of nine individual vibrissae obtained from South American sea lions from Calbuco and Chullec in southern Chile. Black dots represents mean isotopic values of prey. Bars represent standard deviation.

**Fig 5:**
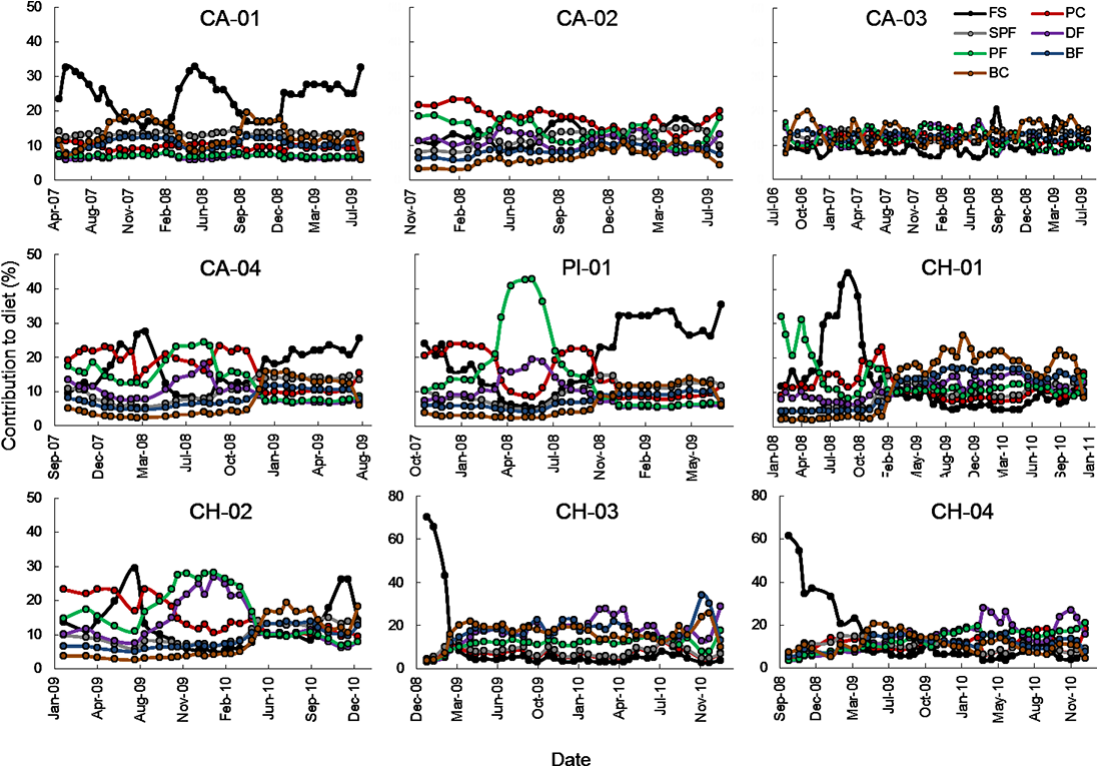
Temporal variation in the median contribution (%) of the seven prey groups to the diet of individual South American sea lions. Vibrissae samples provide a continuum in the diet history of the individual, from the distal sample (farther away from the base) providing the oldest diet information to the proximal sample (closer to the base) representing the most recent diet information. FS: Farmed salmonids, PC: Pelagic cephalopods, SPF: Small pelagic fish, DF: Demersal fish, PF: Pelagic fish, BF: Benthic fish, BC: Benthic crustaceans. Individual IDs are provided in the top of each panel.

The analysis of each individual vibrissae segment within each individual showed a high degree of variability (Fig 4). The median contribution of farmed salmonids fluctuated among segments from 2.8% to 70.4%; both of these estimates were found in CH-02. Salmonids were always low in some sea lion diets (9–10%; CA-02, CA-03), whereas they formed a large component of the diet in others (~24%; CA-01) (Fig 5).

The contribution of salmonids to the diet changed with time. For instance, CH-03 and CH-04 showed high (>50%) consumption of salmonids at the tip of the vibrissa, which we estimate corresponded to late 2008 and early 2009. After this period the contribution of salmon to the diet of these individuals was practically zero (Fig 5). Other individuals showed seasonal cycles in the consumption of salmonids; for example CA-01 showed a higher consumption of this prey in fall and winter months, and a low consumption in spring and summer.

The results of the mixing model using hair and skin data showed that farmed salmonids and benthic crustaceans were the main prey groups consumed by SASL when using hair (median ± SD: 19.7 ± 13.5% for salmonids and 13.3 ± 8.3% for crustaceans), while pelagic fishes and farmed salmonids where the most important prey groups when using skin (median ± SD: 15.3 ± 9.8% and 15.3 ± 9.6% for pelagic fishes and farmed salmonids, respectively) (Table 4). Farmed salmonids were the most important prey group for individuals from Calbuco when using hair data, and from Chullec when using skin data (Table 4). Similar to results for vibrissae, we observed high individual diet variability when using hair and skin data to estimate SASL diet composition. For example, median diet contributions of farmed salmonids derived from hair data varied from as low as 4.7% in CH-01 to as high as 47.6% in CA-04.

**Table 4:** Median contribution (± SD) of the seven prey groups to the diet of SASLs from Calbuco and Chullec by hair and skin data. FS: Farmed salmonids, PC: Pelagic cephalopods, SPF: Small pelagic fish, DF: Demersal fish, PF: Pelagic fish, BF: Benthic fish, BC: Benthic crustaceans.*Indicates significant differences.

Prey group	Hair			Skin		
	Calbuco	Chullec	Mean ± SD	Calbuco	Chullec	Mean ± SD
FS	28.2	9.8 *	19.7 ± 13.5	12.8	19.2	15.3 ± 9.6
PC	11.8	11.5	11.3 ± 5.0	15.1	10.3*	13.1 ± 3.5
SPF	11.8	9.0	10.7 ± 2.8	10.1	12.1	11.1 ± 3.3
DF	6.7	13.4*	9.4 ± 4.1	13.0	9.2	11.3 ± 5.0
PF	7.8	11.8*	9.3 ± 2.6	19.6	9.3	15.3 ± 9.8
BF	8.7	13.6	11.1 ± 4.5	7.6	11.1	9.1 ± 2.9
BC	10.1	15.3	13.3 ± 8.3	5.7	14.0	9.2 ± 6.3

Finally, we found no clear pattern when looking into the relationship between the proportion of time at-sea close to salmon farms with the median contribution of farmed salmon to the diet of SASL (Table 3). For instance, some individuals that consumed a high proportion of salmon (*e*.*g*., CA-06) spent a low proportion of their time within a radius of 1 km from salmon farms.

## Discussion

δ^13^C and δ^15^N values of vibrissae, hair and skin, in conjunction with satellite tracking data indicated that there is (1) spatial overlap between SASL and salmon farms, (2) sea lions are consuming farmed salmon in southern Chile, and (3) there exists substantial inter-individual variability in foraging habits of SASL. While significant interactions have been described between this species and salmon aquaculture in southern Chile [18], [20], our study is the first in any region to link movement patterns of an apex marine predator with the location of aquaculture installations and quantify the amount of farmed salmonids in a native consumer’s diet. Also, the lack of relationship between time spent close to salmon farms and importance of salmon in the diet demonstrated that an integrated approach that combines stable isotope analysis with high-resolution movement data is a powerful means to examine the foraging ecology of a marine predator and characterize its interactions with the environment [16].

### Habitat utilization and spatial interaction with salmon farms

Our study revealed that the most important hot spots for SASL corresponded to the inner and eastern coasts of Chiloé Island in the Gulf of Ancud and the adjacent Reloncaví Fjord. These findings indicate that SASL prefer to forage in the inner coast, despite breeding colonies are on the outer coast. The eastern coast of Chiloe Island is characterized by high densities of pelagic and demersal fishes [61]. In addition, several salmon farms are located at the mouth of Reloncaví Estuary, which is ~50km from the La Sebastiana breeding colony on the outer coast. This distance is well within the foraging range estimated in our and other studies of SASL movement and foraging behavior [62–65]. Thus, sea lions can take advantage of the abundant resources nearby, which could be a beneficial strategy as it confers the advantage of reducing overall travel costs [64].

We found large variability in individual SASL spatial ranges and the degree of overlap with salmon farms. For example, the individual PI-01 showed high use of areas that contained salmon farms. However, salmon was not an important prey item in the diet of this particular individual, accounting for only 8.1% of its diet according to hair isotope data. In contrast, the diet of some individuals that did not show a high spatial overlap with salmon farms (*e*.*g*., CA-01) had a high contribution of salmon to their diet. These results suggest that even if an individual forages in close proximity to salmon farms, they do not necessarily eat farmed salmon. Similar results have been found in other species of pinnipeds that interact with fisheries, in which spatial overlap is not necessarily related with a higher degree of interaction [24], [66]. Even the magnitude of the competition between an apex marine predator and fisheries will depend partly on the degree of spatial and/or temporal resource overlap [67], however, our results suggest that the degree of spatial overlap is not necessarily proportional to the magnitude of interactions.

At least three explanations that are not mutually exclusive could explain the lack of relationship between time spent close to salmon farms and importance of salmon in the diet. First, SASL that spend a significant proportion of time close to salmon farms may actually consume wild native fish that are attracted to the pen enclosures because they provide food or structure [43]. Second, we cannot discard the possibility that SASL are consuming feral salmon that have similar isotopic values as farmed salmon (M. Sepúlveda, unpublished data). High quantities of feral salmon that have escaped from salmon farms are now widely distributed in both marine and freshwater environments in southern Chile [43]. Thus it is possible that SASL may be consuming feral salmon that are not associated with salmon farms. Finally, the results from satellite tracking and SIA data may not match because they are not overlapping in time; obviously tracking data showed the foraging ecology after capture, whereas hair and skin isotopic data are indicative of diet before capture. SASL show a remarkably high degree of intra-individual (within-vibrissae) isotopic variation, which is evidence of prey switching and overall plasticity in foraging behavior. Similar diet plasticity has been observed in other SASL populations [46] and other species of sea lions [1–2], [16], which suggests that this group of marine predators can quickly adapt to spatial and temporal variation in prey availability and abundance.

### Stable isotopes analysis and diet composition

Isotopic data from vibrissae, skin and hair samples showed that farmed salmonids were one of the most important prey for SASL in the study area (Table 4). Although our knowledge of SASL diet composition in southern Chile is limited, our results are consistent with reported evidence of a strong operational interaction between SASL and salmon farms [18], [20]. While interactions have been previously described between other species of sea lions and non-native farmed salmonids, including Australian fur seals (*Arctocephalus pusillus doriferus*) and New Zealand fur seals (*Arctophoca australis forsteri*) [68], our movement and dietary results show that the SASL is capable of adapting to a novel and easily accessible prey, and reinforces the idea that the SASL shows a high plasticity in its trophic habits [22].

Pelagic fish and benthic crustaceans were also identified as important prey sources for SASL. Snoek (*Thyrsites atun*) and Chilean jack mackerel (*Trachurus murphyi*) were important prey species when skin isotope data was used in the mixing model, with a mean group contribution of 15.3 ± 9.8%. Both species have been reported as dietary items for SASL in other areas along the Chilean coast [69], [70]. H. Pavés (unpublished data) also reported a strong interaction between SASL and snoek fisheries at localities close to our study area, which also suggests that this fish species is consumed by SASL. Finally, mixing model results based on hair isotope data identified benthic crustaceans as an important prey group for SASL, accounting for 13.3 ± 8.3%. Crustaceans have been reported as an occasional prey consumed by SASL in Argentina [71] and Chile [72].

Our results showed consistent differences in dietary estimates between hair and skin tissues. Differences in diet composition between tissues could reflect difference in isotopic incorporation rates and thus the time of year that each tissue represents [73–75]. The isotopic composition of skin integrates dietary inputs over relatively recent time scales from 1–3 months prior to sample collection [76]. Thus, our skin samples correspond to the austral winter and spring since they were collected in August and December. Hair remains metabolically inert after formation [75] and since molting in SASL occurs in May–June [77], δ^13^C and δ^15^N values reflect diet consumed during the austral fall. Thus, differences in the isotope values of hair and skin supports seasonal variation in diet composition. Alternatively, the discrepancy in diet composition could be a consequence of the slightly different nitrogen isotope (δ^15^N) trophic discrimination factors (TDFs) used in the mixing models, because results of stable isotope mixing models are sensitive to TDFs [56]. TDFs are known to vary among tissues, a phenomenon often referred to as tissue-specific discrimination. The underlying mechanism for this has not been systematically studied, but differences in amino acid composition among tissues likely play an important role. While we used the same δ^13^C TDF for both tissues, we used a slightly higher δ^15^N TDF for hair (3.5‰) than skin (3.0‰). We performed a sensitivity analysis by running the mixing model using the same δ^15^N TDFs for hair and skin and found that the relative importance of prey identified for each tissue was not sensitive to subtle (0.5‰) differences in δ^15^N TDF.

Independent of whether we use skin of hair data to quantify diet composition via mixing models, our study found evidence of inter-individual variation in prey consumption, especially in the consumption of farmed salmonids, pelagic fish and benthic crustaceans. Isotopic data of SASL tissues collected from Chullec showed differences among individuals and exhibited a larger inter-individual variation in δ^13^C and δ^15^N values as compared to sea lions from Calbuco. The range of SASL δ^15^N values from Chullec suggests that these individuals were foraging on prey from different trophic levels, including a high proportion of benthic crustaceans and benthic fishes. Sea lions from Calbuco, on the other hand, foraged in a narrow isotopic range, showing a high degree of isotopic overlap among the individuals. These inter-individual differences suggest that SASL in some regions may be partitioning available resources that may help reduce intra-specific competition and enable the coexistence of individuals that have overlapping ranges [78]. Similar results have been found in other species of seals [29], [42] and sea lions [28], [79].

We also found a high degree of intra-individual isotopic variation in SASL vibrissae, which was used to quantify variation in diet composition with mixing models at the individual level. Intra-individual changes in diet composition over time likely reflect seasonal changes in the distribution and abundance of prey guilds [1], [2], [16], [66]. For instance, the individual CA-01 showed a clear seasonal signal in the consumption of salmonids with increased consumption of this prey in the fall and winter months (~32%) and corresponding decreases to ~16% in the spring and summer seasons. This pattern corresponds to observational evidence showing that SASL attacks on salmon farms also follow a seasonal pattern, being most prevalent during fall and winter months [20]. This feeding behavior may be driven by the energetic deficit that SASL incur during the summer breeding season (December-March) [18]. Additionally, salmonids represented >60% of the diet of individuals CH-03 and CH-04 in the summer of 2009, however, after that period both of these individuals showed a significant drop to <5% in the consumption of this prey group. This decrease in salmon consumption may have been caused by the mortality that local salmonid farms experienced in late 2008 and early 2009 from the infectious salmon anemia virus [80]. This viral outbreak resulted in the closure of many salmon farms in the region, especially those located on the east coast of Chiloe Island where CH-03 and CH-04 were captured. It is important to note that we cannot discard some errors in our timeline estimates, because we had no species-specific whisker growth rates thus our assumption of 0.16 mm/day found in other otariids [37] may be incorrect.

In summary, our findings demonstrate the usefulness of integrating stable isotope derived dietary data with movement patterns to characterize the impacts of a non-native prey on the foraging ecology of an apex marine predator, and emphasize the importance of a multi-metric approach to understanding predator responses to changes in prey availability [79]. In addition to showing how flexible a generalist predator can be in the face of human alteration of their environment, our results have important applied implications in a situation where interactions between aquaculture and wildlife are common, which has resulted in a call for more active management of SASL in this region. Our results represent an important step in understanding the potential contribution of SASL to salmon mortality and its potential role as a natural regulator of this non-native fish in Chile.

## Supporting Information

S1 Table Individual δ^13^C, δ^15^N and C:N atomic ratio for hair and skin tissues of 12 SASLs from southern Chile. (DOCX)
