# Supplementary material for: Using Satellite Tracking and Isotopic Information to Characterize the Impact of South American Sea Lions on Salmonid Aquaculture in Southern Chile
Source: PLoS One. 2015 Aug 26;10(8):e0134926. doi: 10.1371/journal.pone.0134926 (PMC4550435; doi:10.1371/journal.pone.0134926)
Supplement: S1 Table — (DOCX) [file pone.0134926.s001.docx]

| **ID** | **Hair** | | | **Skin** | | |
| --- | --- | --- | --- | --- | --- | --- |
|  | **δ^13^C** | **δ^15^N** | **C:N** | **δ^13^C** | **δ^15^N** | **C:N** |
| CA-01 | -13.3 | 19.1 | 3.0 | -13.1 | 20.4 | 2.9 |
| CA-02 | -13.3 | 19.1 | ND | - | - | - |
| CA-03 | - | - | - | -13.5 | 20.6 | 3.1 |
| CA-04 | -14.7 | 19.4 | 3.2 | -13.5 | 19.9 | 2.9 |
| CA-05 | -12.5 | 18.4 | 2.3 | -13.0 | 18.6 | ND |
| CA-06 | -12.9 | 17.8 | 2.2 | -13.5 | 18.3 | 2.4 |
| CA-07 | -13.2 | 18.5 | ND | -12.7 | 19.2 | ND |
| PI-01 | -12.0 | 19.0 | 2.3 | -12.7 | 18.2 | ND |
| CH-01 | -11.9 | 19.9 | 2.3 | -12.3 | 18.1 | 2.2 |
| CH-02 | -13.9 | 19.7 | 1.8 | -13.6 | 17.8 | 2.5 |
| CH-03 | -11.7 | 19.4 | 1.7 | - | - | - |
| CH-04 | -12.2 | 19.6 | 2.5 | -12.5 | 19.2 | 2.5 |
| Mean ± SD | -12.9 ± 0.9 | 19.1 ± 0.6 | 2.4 ± 0.5 | -13.0 ± 0.5 | 19.0 ± 1.0 | 2.6 ± 0.3 |

**S1 Table. Individual δ^13^C, δ^15^N and C:N atomic ratio for hair and skin tissues of 12 SASLs from southern Chile.**
